# A 20-year bibliometric analysis of patellofemoral pain syndrome: Current trends and research hotspots (2005–2025)

**DOI:** 10.1097/MD.0000000000050644

**Published:** 2026-09-11

**Authors:** Wen Zheng, Siliang Zeng

**Affiliations:** a Department of Rehabilitation Therapy, Shanghai Normal University Tianhua College, Shanghai, China.

**Keywords:** bibliometric, CiteSpace, hotspots, patellofemoral pain syndrome, visualization, VOSviewer

## Abstract

**Background:**

Patellofemoral pain syndrome (PFPS) is one of the most common and clinically challenging knee disorders and has become a hotspot in orthopedics, sports science, and rehabilitation. The pathogenesis, diagnosis, and treatment of PFPS remain controversial. Global academic studies in PFPS continue to grow, with a steady increase in related research. Although most articles consist of clinical studies or systematic literature reviews, analyses of research hotspots remain limited. This study aims to provide a comprehensive analysis of the current status and research hotspots in PFPS, offering insights for future research and practical applications.

**Methods:**

Publications related to PFPS from 2005 to 2025 were collected from the Web of Science Core Collection database. Data were analyzed and visualized through scientific quantitative and qualitative methods using Microsoft Excel, SPSS Statistics 27, Tableau, CiteSpace, VOSviewer, and Scimago Graphica.

**Results:**

A total of 1022 papers were identified. The United States contributed the most publications (333 articles). The annual number of publications and average citation counts showed an overall increasing trend, with the highest number of articles published in 2022. The article with the highest number of citations was “Patellofemoral pain” written by Willy, RW in 2019, with 182 citations. The *International Journal of Sports Physical Therapy* was the journal that published the most articles on this topic. The University System of Ohio was the most productive institution, and Rathleff, MS was the most published author. Collaborative network analysis showed strong cooperation among researchers from leading countries and institutions. Research hotspots include knee, kinematics, anterior knee pain, hip, biomechanics, reliability, pain, osteoarthritis, strength, and females. The increase in keyword citations appears to be shifting toward clinical applications, including terms such as randomized controlled trials, therapy, and patterns.

**Conclusion:**

Our research has found that the number of PFPS publications and citations has increased significantly since 2019, indicating that the field is moving toward greater complexity. The emerging keywords, such as randomized controlled trial, therapy, and patterns, highlight the shift toward clinical application in this field. This study provides new ideas for scholars and is conducive to further exploration of research directions in this field.

## 1. Introduction

Patellofemoral pain syndrome (PFPS) is one of the most common and clinically challenging knee conditions among adolescents.^[1,2]^ The annual prevalence of patellofemoral pain in the general population is 22.7%, rising to 28.9% in adolescents, with females facing twice the risk of PFPS compared with males.^[3]^ Among athletes, PFPS affects 25% of individuals, with prevalence exceeding 70% in those aged 16 to 25 years.^[4,5]^ PFPS presents as anterior knee pain that is aggravated by physical activities that load the patellofemoral joint.^[6]^ Persistent PFPS can negatively affect social participation as well as athletic or occupational physical performance.^[7]^ The pathogenesis of PFPS remains highly debated and may involve internal factors such as skeletal alignment, soft tissue imbalances, and biomechanical influences, along with external factors like environment and equipment.^[8–10]^ Amid this ongoing discussion, global academic attention in PFPS continues to rise, accompanied by a steady growth in related research. While most articles consist of clinical studies or reviews, there remains a lack of systematic literature reviews and analyses of research hotspots.

Bibliometric analysis is a mathematical and statistical method used to explore bibliometric characteristics.^[11]^ Using visualization tools such as tables and graphs, it can reveal collaboration networks, research components, and emerging trends, thereby helping researchers quickly understand a field, identify research gaps, and gain deeper insights.^[12–14]^ In recent years, bibliometric research has become increasingly important in the medical field.^[15,16]^ However, no studies have yet used bibliometric methods to analyze global research trends on PFPS. Therefore, this study aims to provide a comprehensive analysis of the current status and research hotspots in PFPS over the past 2 decades, and offer suggestions for future research directions and applications.

## 2. Methods

### 2.1. Data collection

**Figure 1.**
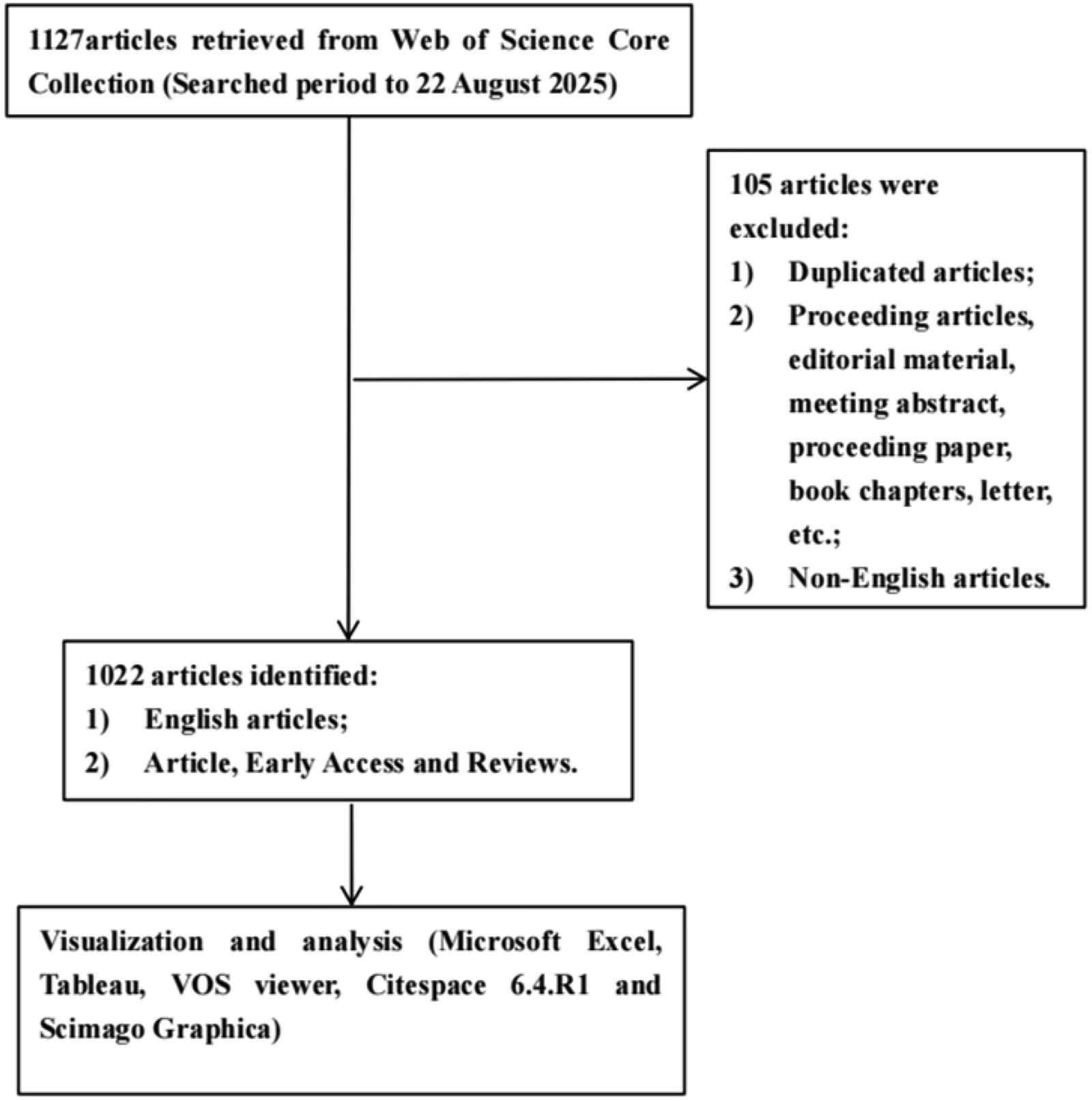
Literature search and screening strategy.

Web of Science stands as one of the world’s authoritative bibliographic databases, holding significant standing within academic research. Consequently, we selected the Web of Science Core Collection (WoSCC) database to enhance data representativeness. Literature types included online articles, research papers, and reviews, and the language was restricted to English. The time span for the database was from the start of the WoSCC database to the present. The indices included SCI-Expanded, SSCI, A&HCI, CPCI-S, CPCI-SSH, BKCI-S, BKCI-SSH, ESCI, CCR-Expanded, and IC. The search terms were developed using the Medical Subject Headings term “Patellofemoral Pain Syndrome” in PubMed, together with its related free-text terms and synonyms. These terms were subsequently searched in the WoSCC using the Topic (TS) field. The detailed search strategy was as follows: TS = (“Patellofemoral Pain Syndrome” OR “Pain Syndrome Patellofemoral” OR “Anterior Knee Pain Syndrome” OR “Patellofemoral Syndrome” OR “Patellofemoral Pain” OR “Pain Patellofemoral” OR “Patellofemoral Pains”). To minimize errors arising from frequent database updates, all literature retrieval and downloading procedures were completed within 1 day on August 22, 2025. Following screening, a total of 1022 articles were ultimately included, subsequently exported as records in “plain text” format and as “full records and references.” The literature retrieval and screening process is illustrated in Figure 1. Data standardization was performed: keyword fields were standardized by unifying parts of speech and the singular and plural forms (e.g., exercises and exercise merged to exercise) to reduce meaningless duplication in keyword clustering. Country/region standardization was also applied (e.g., England, Scotland, Wales, and Northern Ireland were merged as the United Kingdom) to ensure data consistency and comparability.

### 2.2. Statistical analysis

We first extracted basic characteristics of the literature from the WoSCC literature analysis report. Then, we performed multidimensional analysis using Microsoft Excel (Microsoft Corporation), Tableau (Tableau Software, LLC), CiteSpace (version 6.4 R1), VOSviewer, Scimago Graphica, and SPSS Statistics version 27 (IBM Corporation). Among these, CiteSpace, developed by Professor Chen Chaomei’s team, applies citation analysis theory and pathfinding network algorithms to study co-citation relationships and keyword trends over time. In this study, CiteSpace was used to analyze co-occurrence networks, detect emerging terms, and display timeline distributions of co-cited references and keywords. VOSviewer, a Java-based tool created by Professors van Eck and Waltman, is commonly used to build collaboration and co-occurrence networks.^[13,17,18]^ In this study, VOSviewer was used to visualize collaborations among countries, institutions, and authors, as well as keyword clusters and co-occurrence relationships. We also collected the data for the country collaboration network using VOSviewer and exported it in gml. format. The exported data were then used to redraw the country collaboration network diagram with Scimago Graphica. SPSS Statistics 27 was used to compute Spearman correlation coefficients to evaluate relationships between ordinal variables. A *P* value <.05 was considered statistically significant, and a larger absolute value of ρ indicated a stronger correlation between the 2 variables.

## 3. Results

### 3.1. Overview of published literature

**Figure 2.**
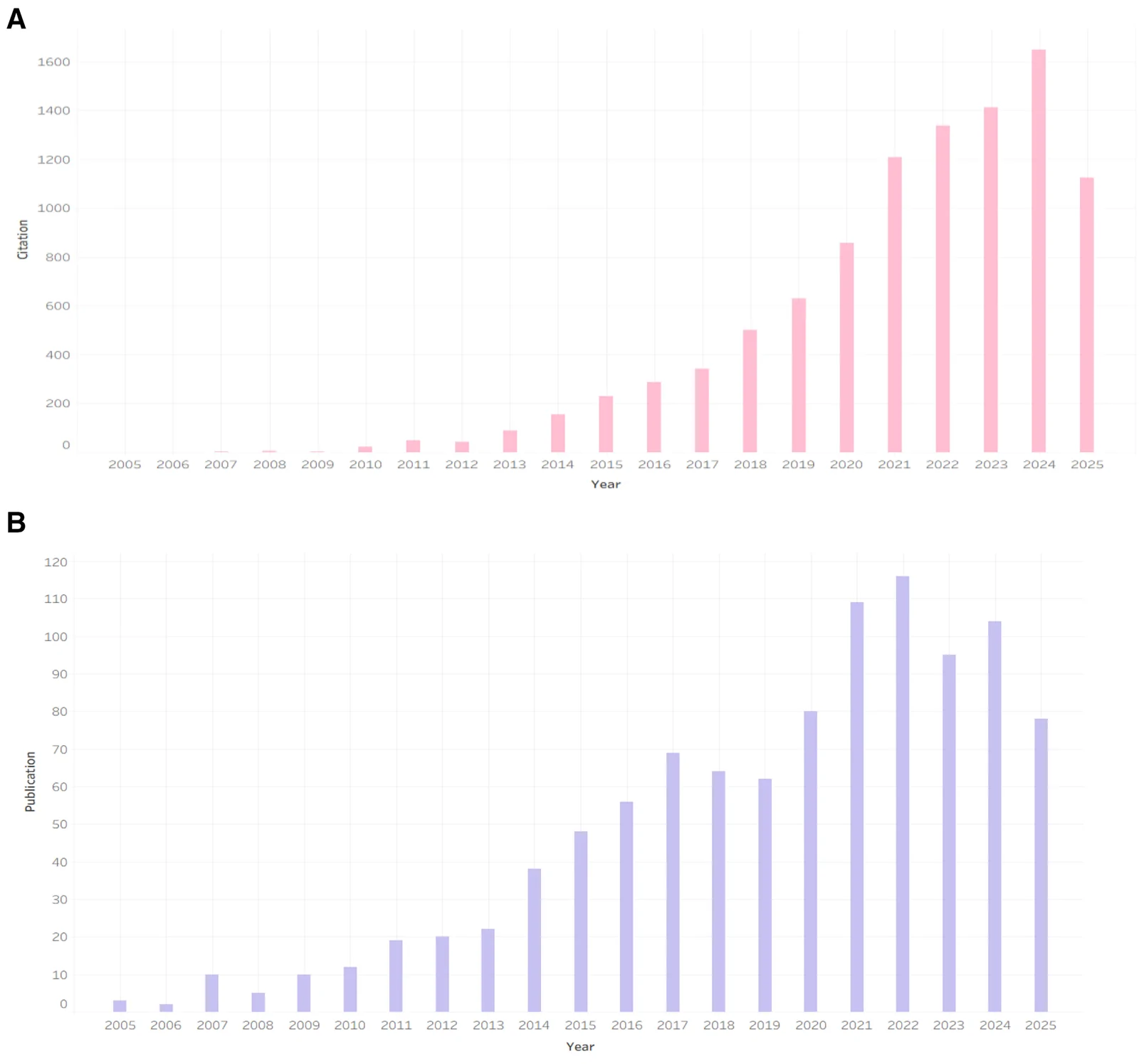
(A) The number of articles regarding PFPS published annually. (B) The frequency of citations of articles regarding PFPS published annually. PFPS = patellofemoral pain syndrome.

This study included a total of 1022 articles, consisting of 890 articles and 132 reviews. The annual number of publications and citations reflects the development of the research field and current trends. Figure 2A shows the annual number of articles on PFPS, and Figure 2B presents the annual citation counts. Both show an overall increasing trend, with a substantial rise since 2019. The highest number of articles was published in 2022 (116 articles), while 2024 received the most citations (1649), with an average of 9.72 citations per article. We used Spearman correlation analysis to explore the relationship between annual publication count and citation frequency. The results showed a strong positive correlation (ρ = 0.974, *P* < .001), indicating that as the number of publications increases, so does the number of citations, and vice versa.

**Figure 3.**
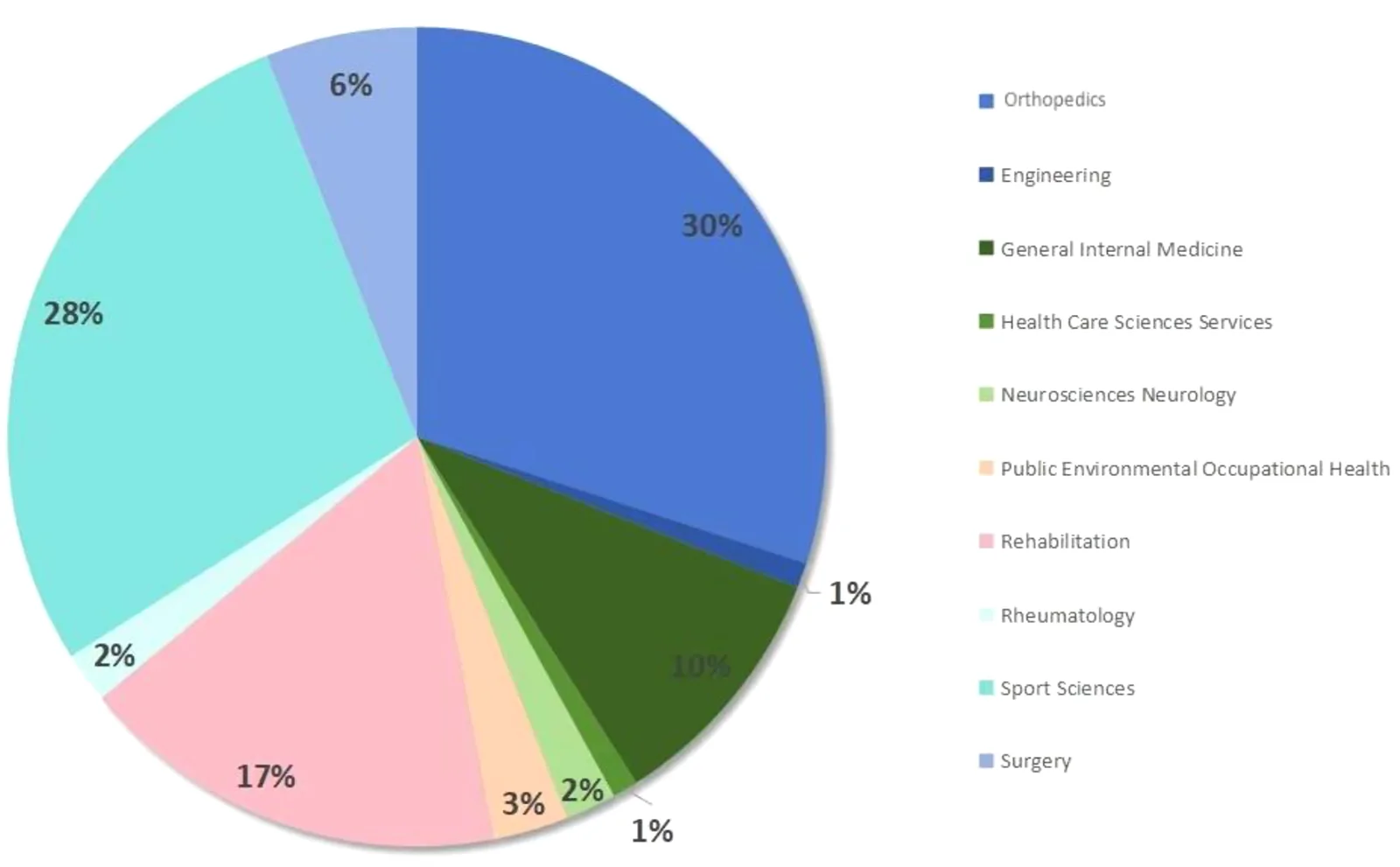
Research areas of publications.

Analysis of research fields and disciplines helps reveal interdisciplinary connections and disciplinary dynamics, providing quantitative support for academic research and research management. As shown in Figure 3, the top 3 published research areas of PFPS are Orthopedics, Sport Sciences, and Rehabilitation, followed by General Internal Medicine, Surgery, Public Environmental Occupational Health, Neurosciences Neurology, Rheumatology, Engineering, and Health Care Sciences Services.

### 3.2. Cooperative relationship

By analyzing the publication output of different countries/regions, institutions, and authors, along with impact metrics such as citation counts, it is possible to identify co-occurrence relationships and high-impact collaborative partnerships, thereby clarifying the structure and logic of academic collaboration networks. As shown in Table 1, the top 10 countries and funding agencies in the field of PFPS are presented. The United States ranks first with 333 publications and 4364 citations. Among the funding agencies, the National Institutes of Health (21 publications), the United States Department of Health and Human Services (21 publications), the National Institutes of Health Research Council (9 publications), and the National Institute of Arthritis and Musculoskeletal and Skin Diseases (7 publications) are all from the United States. It is worth noting that although India ranks relatively high in terms of annual publication output, its citation count ranks the lowest.

**Table 1:** The top 10 most productive countries and funding agencies in the PFPS research field.

Top 10 countries in the PFPS area			Top 10 fund agencies of the article	
Country	Publication	Citation	Fund agency	Count
USA	333	4364	National Institutes of Health	21
England	93	1122	United States Department of Health Human Services	21
Iran	71	542	Coordenacao De Aperfeicoamento De Pessoal De Nivel Superior	20
Australia	65	1323	Shiraz University of Medical Science	18
India	64	123	National Health Medical Research Council of Australia	12
Brazil	63	347	Conselho Nacional De Desenvolvimento Cientifico E Tecnologico	11
Canada	41	477	National Institutes of Health Research Council	9
Spain	40	392	Canadian Institutes of Health Research	8
Italy	38	285	Fundacao De Amparo A Pesquisa Do Estado De Sao Paulo	7
China	36	345	National Institute of Arthritis and Musculoskeletal and Skin Diseases	7

PFPS = patellofemoral pain syndrome.

**Figure 4.**
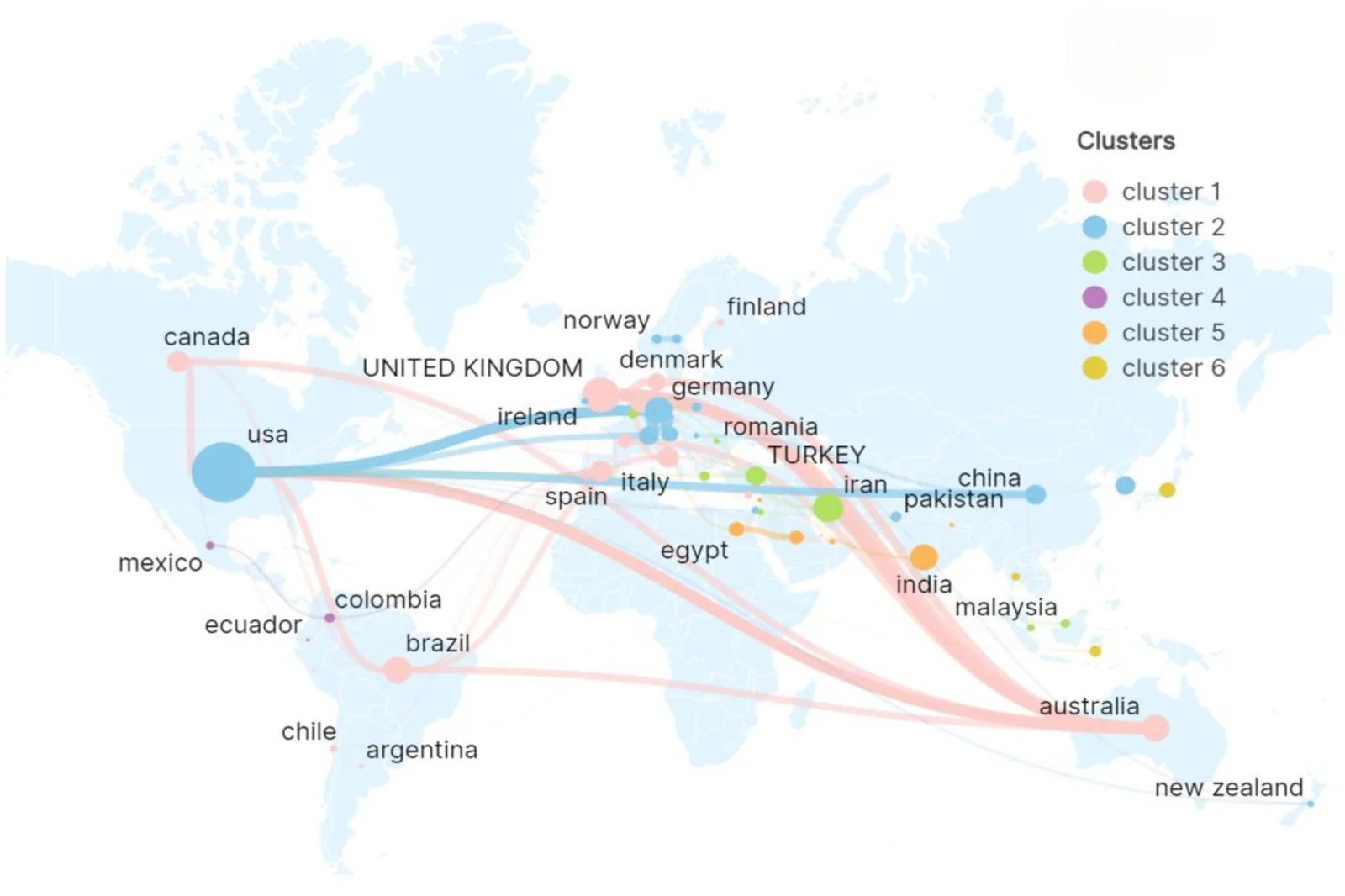
Cooperation among countries/regions participating in the research.

A total of 66 countries worldwide have participated in PFPS research. We performed clustering analysis on 38 countries with more than 5 publications each and visualized the results with Scimago Graphica by VOSviewer. As shown in Figure 4, the United States, the United Kingdom, Australia, Brazil, Italy, Spain, Canada, China, and other countries form close collaborative connections, which highly align with the top 10 countries in terms of publication output.

**Figure 5.**
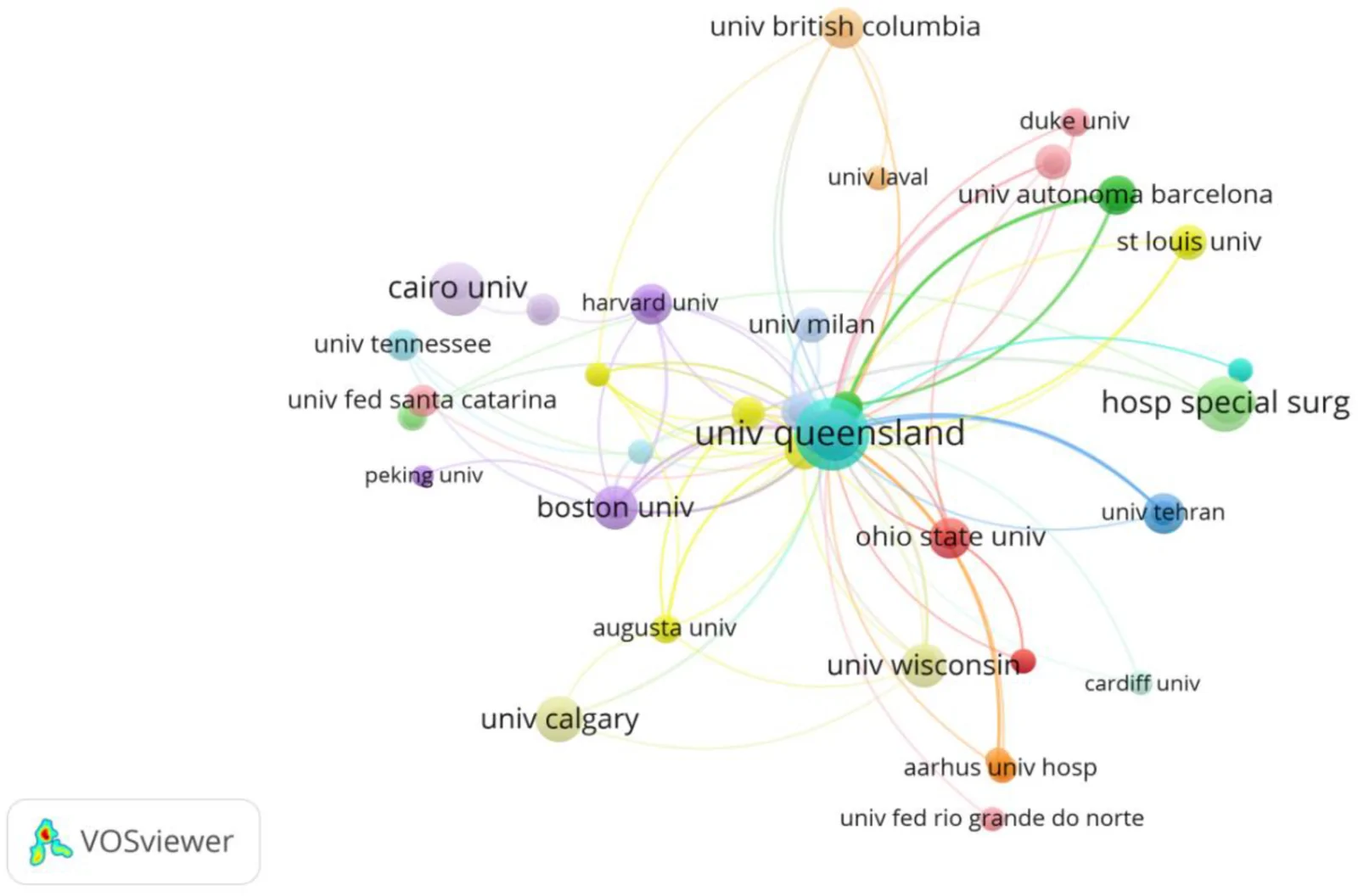
Cooperation network diagram of research institutions in the PFPS field. PFPS = patellofemoral pain syndrome.

Table 2 lists the top 10 most productive institutions, 4 of which are from the United States. The institution with the highest number of publications is the University System of Ohio (28 articles). The collaboration network among institutions is shown in Figure 5. The University of Queensland appears as a larger node in the collaboration network, indicating that most institutional collaborations are centered around it.

**Table 2:** The top 10 research institutions in the PFPS research field.

Affiliation	Country	Count
University System of Ohio	USA	28
University of Queensland	Australia	23
La Trobe University	Australia	22
Harvard University	USA	21
Egyptian Knowledge Bank	Egypt	20
Harvard University Medical Affiliates	USA	16
University of London	England	16
Aalborg University	Denmark	15
Cairo University	Egypt	15
Hospital for Special Surgery	USA	15

PFPS = patellofemoral pain syndrome.

**Figure 6.**
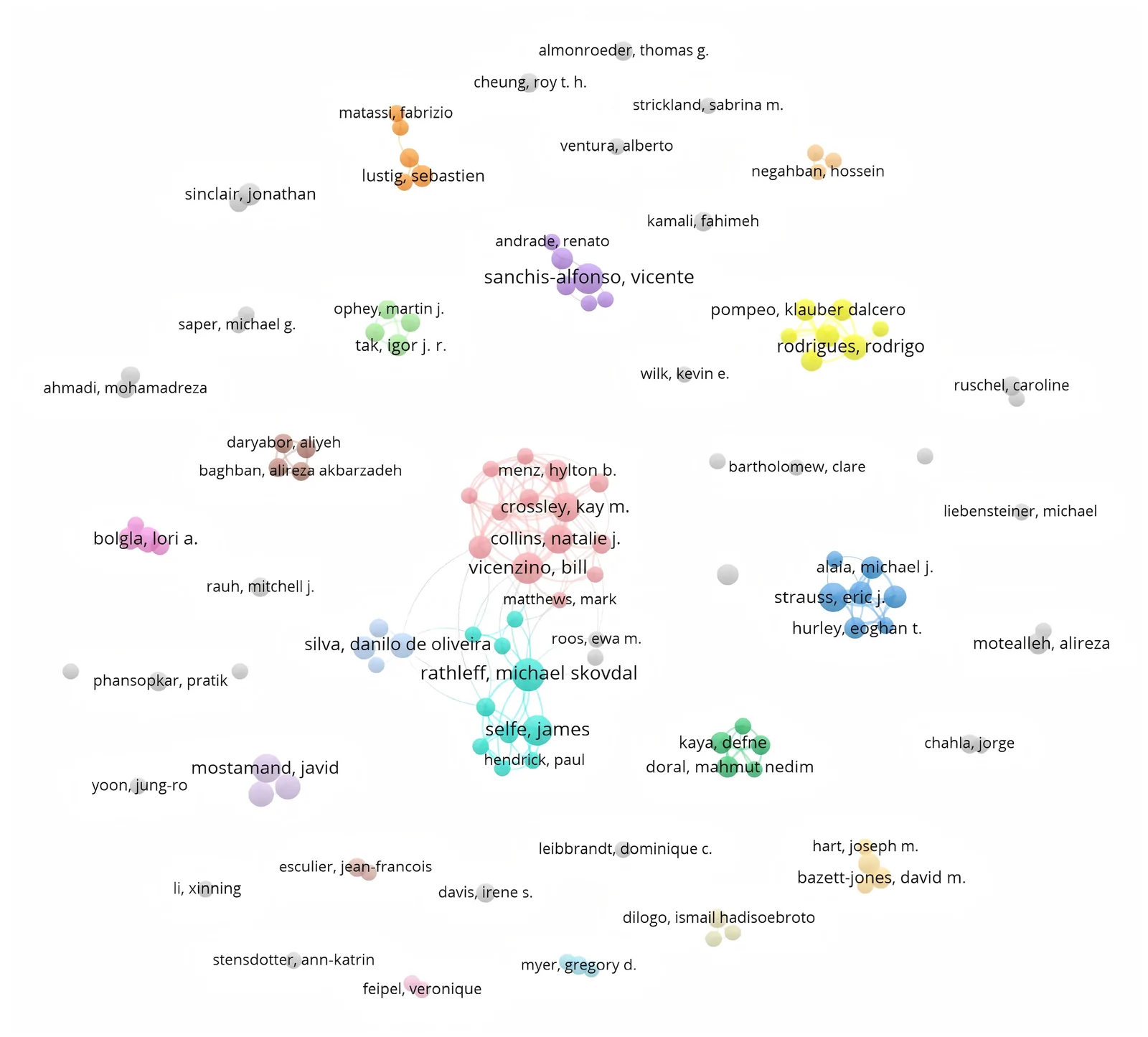
Author collaboration network in the PFPS field. PFPS = patellofemoral pain syndrome.

Table 3 lists the top 10 authors by number of publications, and Figure 6 shows the collaboration network of authors with more than 4 publications. Among the top 10 authors, 2 closely connected collaborative groups have formed, and these 2 groups are further interconnected through other authors to form a larger collaborative network. Rathleff, MS, and Selfe, J show strong collaboration, while Vicenzino, B, Collins, NJ, and Crossley, KM (all from Australia) also exhibit close cooperation. The *H*-index, calculated according to the John-Hirsch method, indicates that a journal or researcher has published at least *H* papers, each of which has been cited at least *H* times, highlighting influential researchers and potential collaborators.^[19]^ Among the top 10 authors, 6 have an *H*-index exceeding 40, indicating that these scholars possess considerable academic influence and high-quality research output in the field of PFPS research.

**Table 3:** The top 10 authors in the PFPS research field.

Author	Country	Institution	*H*-index	Publication
Rathleff, MS	Denmark	Aalborg University	41	13
Vicenzino, B	Australia	The University of Queensland	68	11
Monllau, JC	Spain	Pompeu Fabra University	33	10
Collins, NJ	Australia	The University of Queensland	41	9
Crossley, KM	Australia	La Trobe University	75	9
Mostamand, J	Iran	Isfahan University of Medical Sciences	8	9
Sanchis-Alfonso, V	Spain	Hospital Universitari Arnau Vilanova	21	9
Selfe, J	USA	New York University Langone Orthopedic Hospital	44	9
Strauss, EJ	England	Manchester Metropolitan University	17	9
Bader, DL	England	University of Southampton	48	7

PFPS = patellofemoral pain syndrome.

### 3.3. Active journals

**Figure 7.**
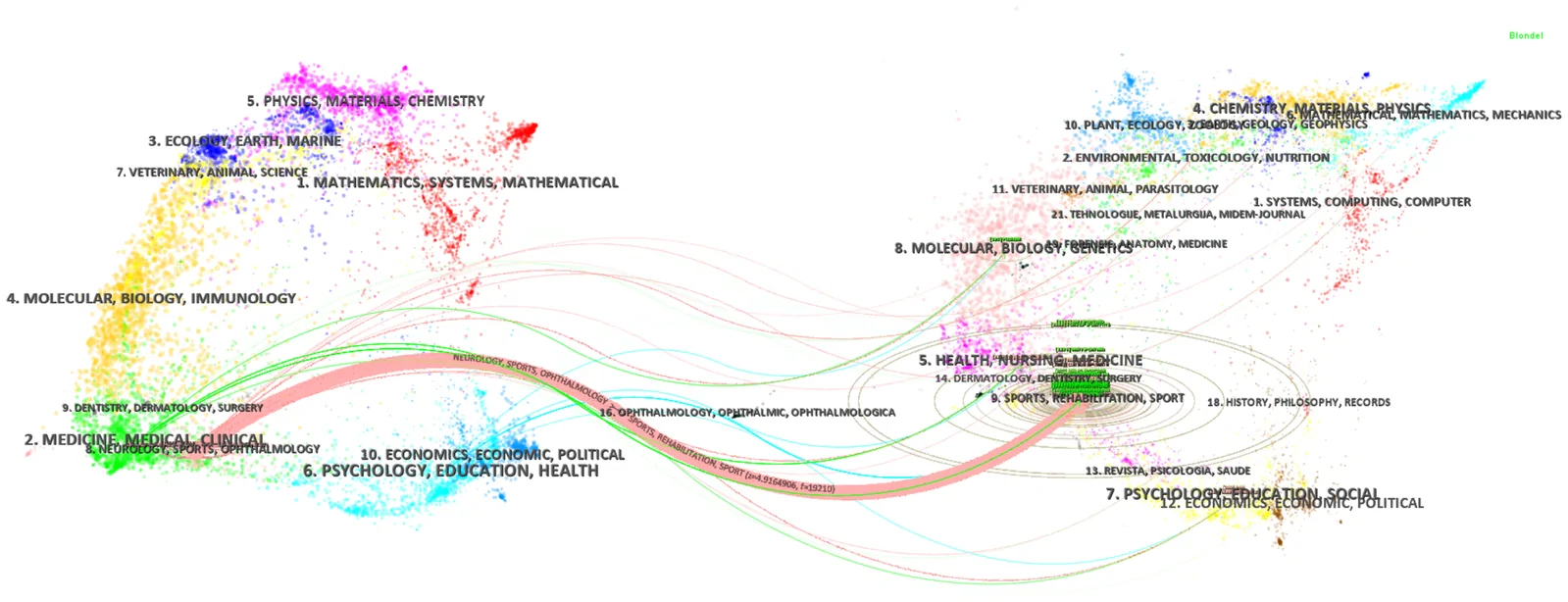
Dual-overlay map in the field of PFPS. PFPS = patellofemoral pain syndrome.

A total of 285 academic journals contributed to research on PFPS in this study. Table 4 lists the top 10 journals by publication counts. Among these, 3 are from the United States, and Orthopedics, Sport Sciences, and Rehabilitation have the largest number of PFPS publications. Journal citation reports provides comprehensive journal evaluation metrics, categorizing journals within the same discipline into quartiles: the top 25% are classified as Q1, the next 25% to 50% as Q2, and so on. The majority of the top 10 journals are ranked as Q1 or Q2. The dual-overlay map of journals in Figure 7 illustrates citation relationships and interdisciplinary connections among research topics. The left side represents journals citing others, while the right side shows journals being cited. Labels indicate the disciplines covered by the journals, and colored paths denote citation links. Journals in Sports, Rehabilitation, and Sport are primarily cited by those in Medicine, Medical, and Clinical fields, demonstrating the interdisciplinary characteristics of PFPS research.

**Table 4:** Top 10 journals with the highest frequency in the field of PFPS.

Rank	Journal	Publications	Country	Citation (WOS)	IF (2024)	Categories	Quartile
1	*International Journal of Sports Physical Therapy*	83	USA	3620	2.1	Sport Sciences	Q2
2	*Journal of Bodywork and Movement Therapies*	68	England	4145	1.4	Rehabilitation	Q3
3	*Cureus Journal of Medical Science*	40	USA	67,722	1.3	Medicine, General & Internal	Q2
4	*Arthroscopy Techniques*	33	Netherlands	3369	1.1	Orthopedics	Q3
5	*Journal of Isakos Joint Disorders Orthopaedic Sports Medicine*	26	England	4145	1.4	Orthopedics Sport Sciences	Q1 Q1
6	*Disability and Rehabilitation*	17	England	18,119	2.0	Rehabilitation	Q2
7	*Journal of Experimental Orthopaedics*	17	Germany	1911	2.7	Orthopedics Surgery	Q2 Q1
8	*International Journal of Environmental Research and Public Health*	16	Switzerland	123,105	4.6 (2021)	Environmental Sciences Public, Environmental & Occupational Health	Q2 Q1
9	*Journal of Orthopaedics*	16	India	3160	1.5	Orthopedics	Q3
10	*Current Orthopaedic Practice*	13	USA	371	0.3	Orthopedics	Q4

IF = impact factor, PFPS = patellofemoral pain syndrome, WOS = Web of Science‌.

### 3.4. Analysis of cited references

Academic influence can be quantitatively assessed by metrics such as total citations and *H*-index, although the impact of factors such as excessive self-citations cannot be entirely excluded.^[20]^ In this field, after excluding self-citations, the total number of citations is 6480, and the *H*-index is 44. Citation analysis serves as an effective method for identifying core knowledge within a specific research network.^[21]^ As shown in Table 5, the top 10 most cited publications in the field of PFPS include 6 research articles and 4 review articles. The most frequently cited paper is a clinical guideline on PFPS authored by Willy, RW, published in the *Journal of Orthopaedic & Sports Physical Therapy*, which has been cited 182 times.

**Table 5:** The top 10 cited articles in the PFPS research field.

Rank	Title	Citation	First author	Journal	Year
1	Patellofemoral pain	182	Willy, RW	*Journal of Orthopaedic & Sports Physical Therapy*	2019
2	Is knee pain during adolescence a self-limiting condition? Prognosis of patellofemoral pain and other types of knee pain	159	Rathleff, MS	*American Journal of Sports Medicine*	2016
3	A systematic review of running-related musculoskeletal injuries in runners	141	Kakouris, N	*Journal of Sport and Health Science*	2021
4	The psychological features of patellofemoral pain: a systematic review	137	Maclachlan, LR	*British Journal of Sports Medicine*	2017
5	Incidence and prevalence of musculoskeletal injury in ballet: a systematic review	134	Smith, PJ	*Orthopaedic Journal of Sports Medicine*	2015
6	Biomechanics and pathomechanics of the patellofemoral joint	124	Loudon, JK	*International Journal of Sports Physical Therapy*	2016
7	Changes in catastrophizing and kinesiophobia are predictive of changes in disability and pain after treatment in patients with anterior knee pain	113	Doménech, J	*Knee Surgery Sports Traumatology Arthroscopy*	2014
8	Is virtual reality effective in orthopedic rehabilitation? A systematic review and meta-analysis	106	Gumaa, M	*Physical Therapy*	2019
9	The patellofemoral pain and osteoarthritis subscale of the KOOS (KOOS-PF): development and validation using the COSMIN checklist	104	Crossley, KM	*British Journal of Sports Medicine*	2018
10	ACL rehabilitation progression: where are we now?	100	Cavanaugh, JT	*Current Reviews in Musculoskeletal Medicine*	2017

ACL = anterior cruciate ligament, PFPS = patellofemoral pain syndrome.

**Figure 8.**
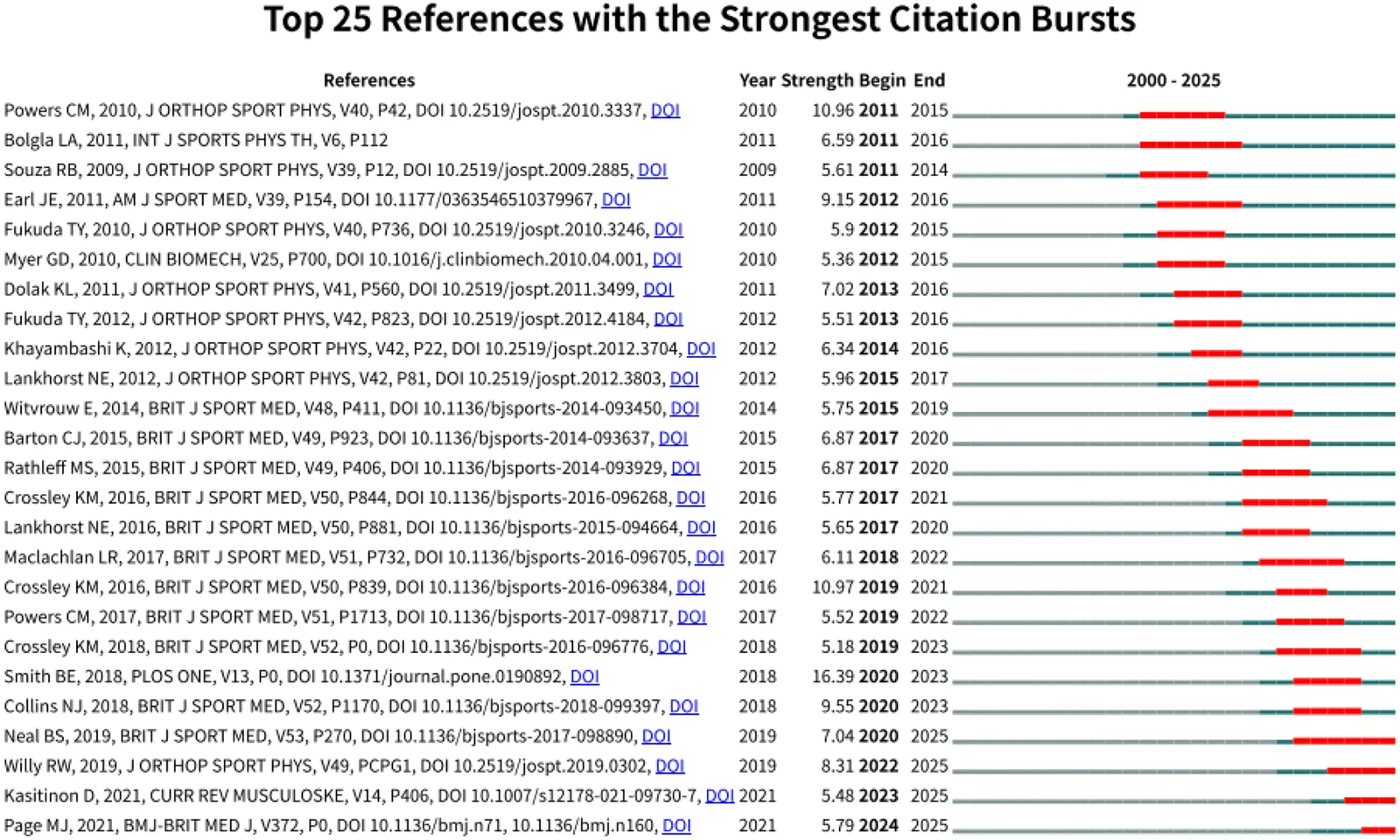
PFPS citation burst reference list. PFPS = patellofemoral pain syndrome.

“Citation burst references” refer to articles that experience a sharp increase in citations shortly after publication, helping to identify influential literature within a specific period. In Figure 8, the blue line represents the timeline, and the red segments on the timeline indicate detected bursts (showing the start year, end year, and duration of the burst). Three articles exhibited a burst strength of >10, indicating they are among the top 3 most frequently cited publications. From 2019 to 2021, the article titled, “Incidence and prevalence of patellofemoral pain: a systematic review and meta-analysis” had a burst strength of 16.35. Between 2011 and 2015, “The influence of abnormal hip mechanics on knee injury: a biomechanical perspective” reached a burst strength of 10.95. From 2019 to 2021, “2016 patellofemoral pain consensus statement from the 4th International Patellofemoral Pain Research Retreat, Manchester” showed a burst strength of 10.94.

### 3.5. Co-occurrence keywords and burst keywords

**Figure 9.**
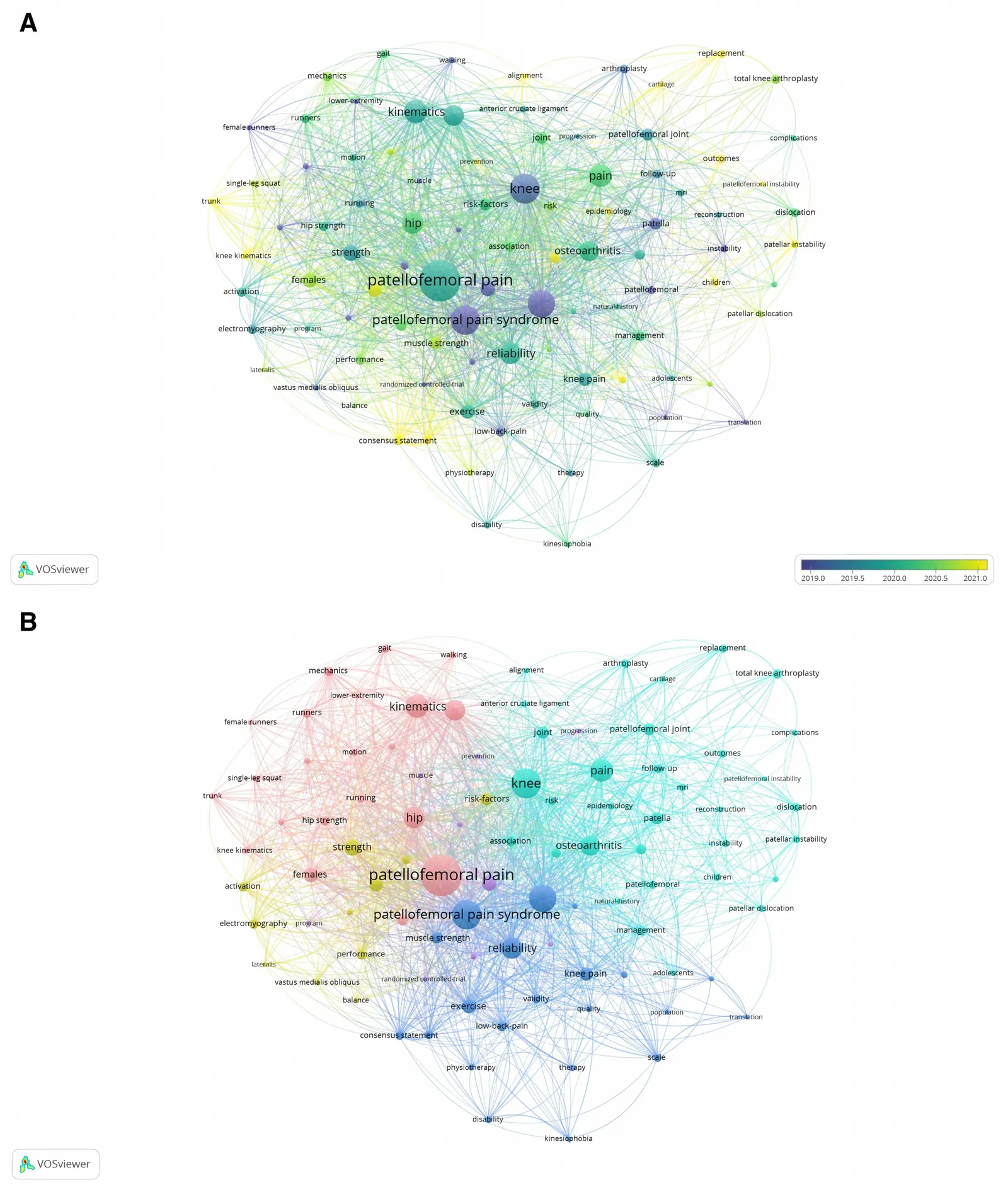
(A) Overlay visualization of keywords. (B) Cluster network map. Nodes of the same color form a cluster.

Keywords represent the main research content of publications and reflect research hotspots and emerging trends in a field. Among the 3320 keywords identified, which include both author keywords and supplementary keywords, an overlay visualization was performed for the top 110 keywords, as shown in Figure 9A. In this figure, higher-quality articles are represented by larger labels and circles. The color of each node is determined by its average publication year. Lines between nodes indicate correlation links, and the color bar at the bottom right, ranging from blue to yellow, represents the timeline from earlier to more recent. Ranked by centrality, aside from the 3 most frequent keywords (patellofemoral pain, patellofemoral pain syndrome, and anterior knee pain), the top keywords in order are knee (occurrences: 185), kinematics (occurrences: 128), pain (occurrences: 125), reliability (occurrences: 117), hip (occurrences: 115), biomechanics (occurrences: 110), osteoarthritis (occurrences: 103), strength (occurrences: 77), females (occurrences: 67), and rehabilitation (occurrences: 67). These have been identified as current research hotspots. Figure 9B shows a cluster visualization of keywords attributed to different clusters. In the main clusters, the green cluster focuses on the pathogenesis, diagnosis, and surgical treatment of PFPS, including terms such as replacement, total knee arthroplasty, and reconstruction. The blue cluster emphasizes conservative treatment and functional rehabilitation, including muscle strength, physiotherapy, and exercise. The pink cluster represents biomechanical aspects of PFPS, containing keywords such as kinematics, mechanics, and motion. Other smaller clusters, such as the yellow cluster, concentrate on the neurophysiology of PFPS, including terms like electromyography, activation, and performance.

**Figure 10.**
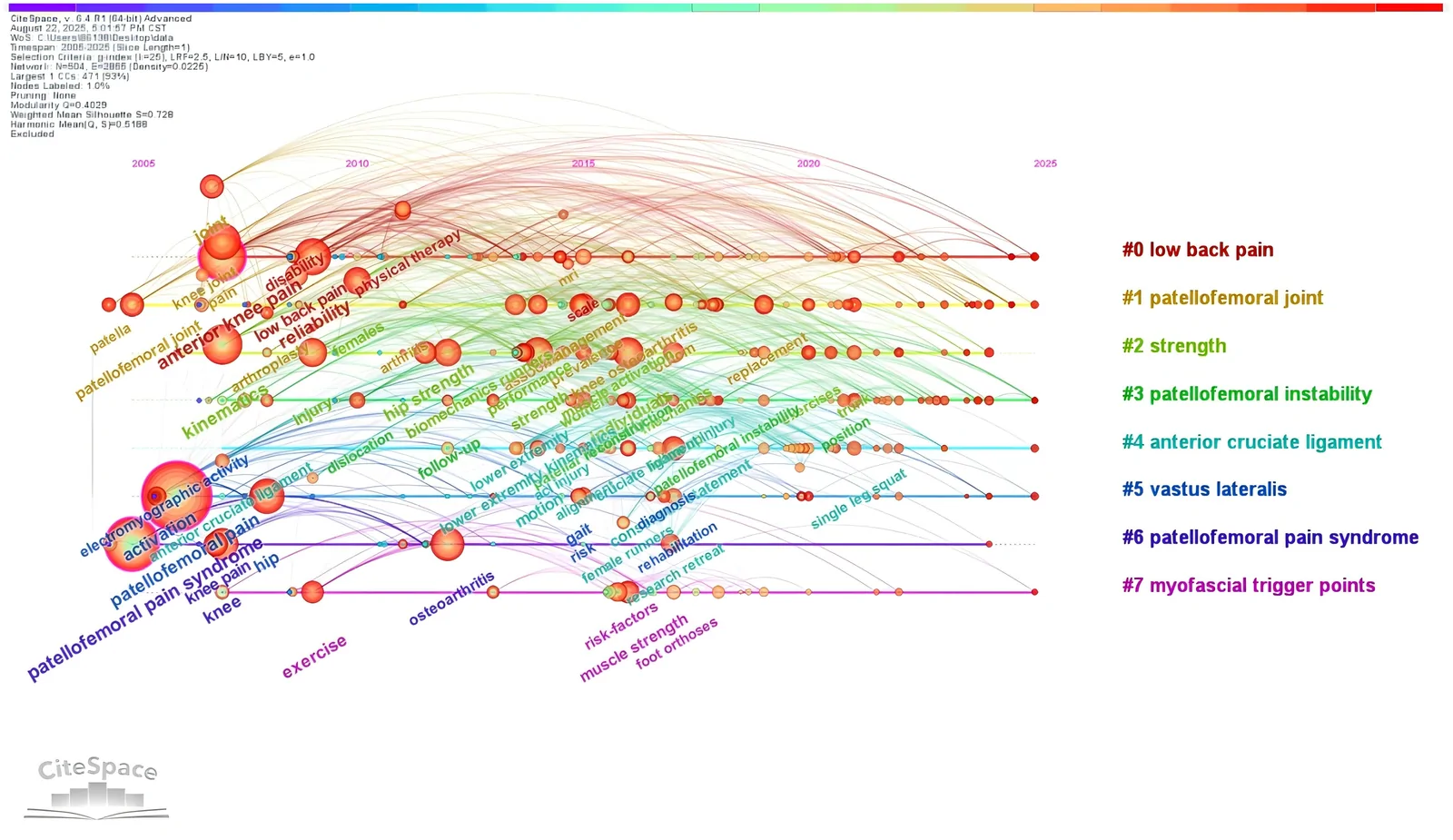
Timeline viewer of keyword evolution.

Visualization of stage-specific research hotspots was conducted using the timeline viewer in CiteSpace software (as shown in Fig. 10). In the early stage (2005–2009), studies primarily focused on the area surrounding the knee joint, with major keywords including patella, knee joint, and anterior cruciate ligament. In the subsequent period (2010–2019), research expanded into more related domains, incorporating topics such as physical therapy, replacement, and rehabilitation, which pertain to postoperative rehabilitation of PFPS, as well as lower extremity kinematic and gait, reflecting a focus on the kinetic chain of the lower limb. More recent research (2020–2025) has further diversified and become more specialized, demonstrating an extension of research directions. Keywords such as exercise, position, and single-leg squat highlight the evolving emphasis on functional recovery in PFPS.

**Figure 11.**
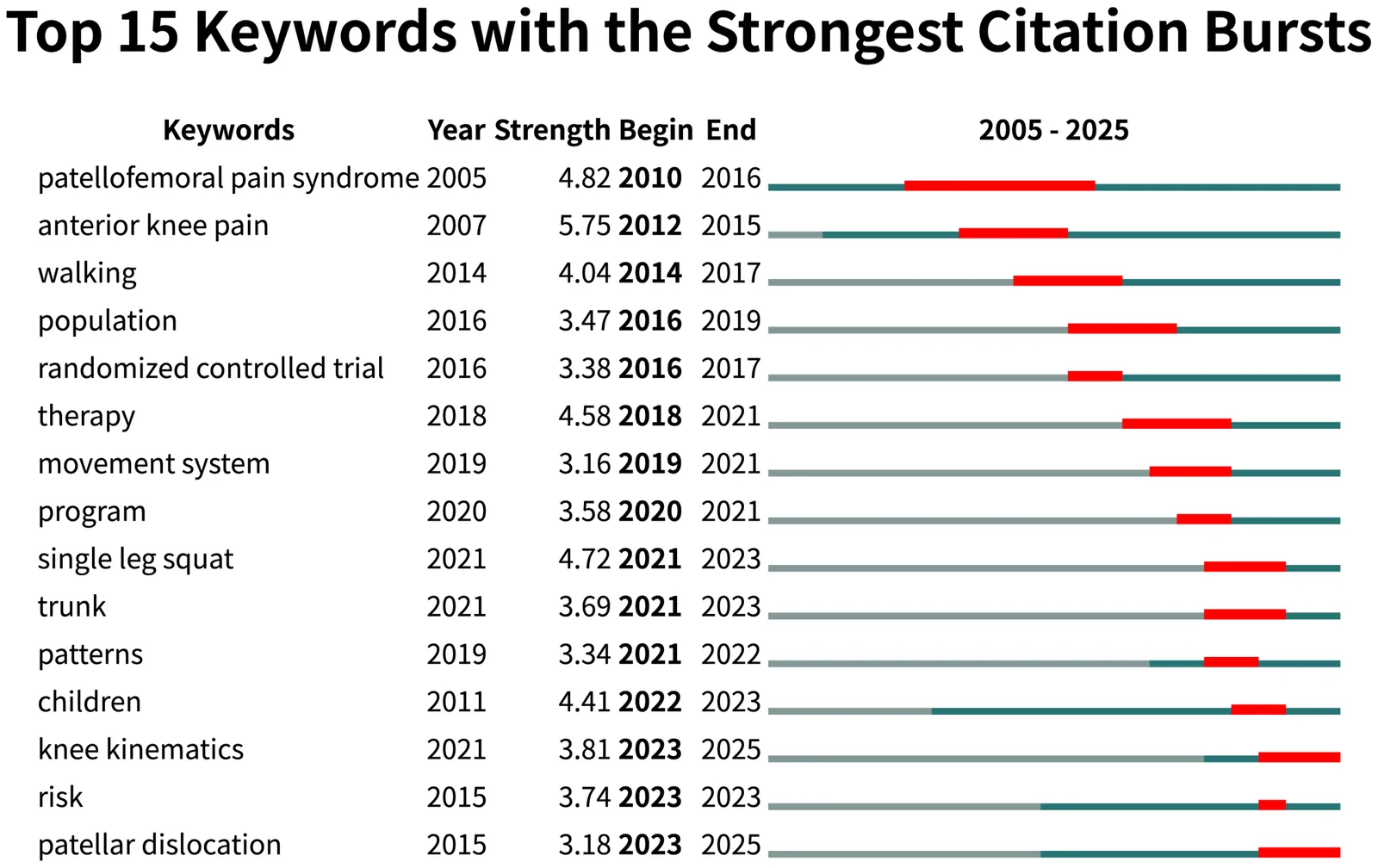
Top 15 keywords with the strongest citation bursts in the PFPS field from 2005 to 2025. PFPS = patellofemoral pain syndrome.

Keyword citation bursts refer to a sharp increase in the frequency of specific keywords in published articles, which can be used to identify frontier topics in a particular period. Figure 11 shows that “anterior knee pain” had the highest citation burst strength (5.75), followed by “patellofemoral pain syndrome” (4.82), “single leg squat” (4.72), “therapy” (4.58), and “children” (4.41). Keywords currently still in a burst phase include “knee kinematics” and “patellar dislocation.”

## 4. Discussion

### 4.1. Global research trends in PFPS

The annual variation in the number of articles related to PFPS clearly reflects the development and trends in academic output in this field. We observed a significant global increase in both the pace and volume of PFPS research, highlighting its growing academic attention. The earliest study on PFPS in the WoSCC dates back to 2005, but a notable surge in publications and citations occurred around 2019, likely influenced by the clinical practice guidelines for PFPS related to the International Classification of Functioning, Disability, and Health, published by Willy, RW et al^[22]^ on behalf of the American Physical Therapy Association. Currently, approximately 49 articles on PFPS are published each year, with Orthopedics, Sport Sciences, and Rehabilitation being the dominant disciplinary categories.

The national collaboration network reveals clear disparities between developed and developing countries in PFPS research. Among the top 10 countries by publication output, 6 are developed nations: the United States, the United Kingdom, Australia, Canada, Spain, and Italy. The United States leads with 333 articles, and most international collaborations center around American institutions, which may be attributed to factors such as national gross domestic product, healthcare funding, advanced medical facilities, demographic factors, and disease incidence. As indicated in Table 1, 4 of the top 10 funding agencies are based in the United States. It is worth noting that although India ranks relatively high in annual publication count, its citation count is the lowest among leading countries, likely because a large proportion of Indian publications were published in 2023 to 2024.

A total of 4158 authors have contributed to PFPS research. The most productive author is Rathleff, MS, with 13 publications, while the tenth-ranked author, Bader, DL, has 7 publications. In addition, 7 authors have published fewer than 10 articles each. The author collaboration network appears relatively fragmented, with most cooperation occurring within the same country, and international collaboration remaining limited. These findings suggest that the PFPS research community is still relatively small and emerging, with some sub-research directions having formed, but the core research teams have yet to expand substantially. This emphasizes the need to broaden research efforts, enhance communication among different teams, and promote vigorous development in the field.

In PFPS research, 285 academic journals have published relevant studies. As shown in Table 4, the top 10 journals by publication count are primarily in Orthopedics, Sport Sciences, and Rehabilitation, with 6 journals ranked in the Q1 or Q2 categories. The most active journal is the *International Journal of Sports Physical Therapy* (from the United States), which has published 83 articles and received 3620 citations, indicating its high recognition among researchers. Given the complexity of PFPS, the field is relevant not only to orthopedics, sports science, and rehabilitation but also to rheumatology, public environmental occupational health, and health care sciences services. Therefore, future researchers should engage in multidisciplinary collaboration to facilitate integration and progress across these disciplines.

### 4.2. Analysis of research hotspots and current state

Among the top 10 most productive authors, 3 main research directions are psychological factors and nonsurgical interventions, biomechanics and physical therapy, and surgical treatment. Notably, unlike most researchers focused on PFPS treatment, Sanchis-Alfonso, V primarily investigates the correlation between PFPS and hip joint disorders. This offers a potential research direction for future studies – by comparing hip structure and function between PFPS patients and healthy individuals, the relationship between hip disorders and PFPS can be further analyzed, promoting a holistic perspective in musculoskeletal disease research.

Among the top 10 most cited references, 6 are research articles, and 4 are review articles, all published after 2015. The most cited article is a clinical practice guideline related to PFPS and the International Classification of Functioning, Disability, and Health, published by the Orthopaedic Section of the American Physical Therapy Association in the *Journal of Orthopaedic & Sports Physical Therapy*, with 182 citations. Among these highly cited works, 2 focus on clinical guidelines and prognosis studies, providing evidence-based recommendations for diagnosis and intervention in PFPS clinical management, serving as core references for practice.^[22,23]^ Two publications cover biomechanics, pathological mechanisms, and assessment tools of PFPS, analyzing its pathogenesis, such as patellofemoral joint reaction forces and abnormal tracking – from anatomical and mechanical perspectives.^[24]^ Crossley, KM et al^[25]^ developed a new subscale, the Patellofemoral Pain and Osteoarthritis Outcome Score, enhancing standardization in both research and clinical practice. Three articles focus on psychological factors and rehabilitation interventions in PFPS,^[26–28]^ and 2 examine epidemiology in specific populations such as runners and ballet dancers.^[29,30]^ One article on anterior cruciate ligament rehabilitation, although less directly related to PFPS, represents an important direction in knee rehabilitation and shares some common ground with PFPS in rehabilitation principles.^[31]^

Citation bursts reflect publications that were frequently cited within specific time periods. From 2011 to 2015, Powers, CM et al^[32]^ proposed that addressing proximal dysfunction related to knee injuries could improve pelvic/trunk stability and dynamic hip control. Between 2012 and 2016, Earl, JE et al^[33]^ demonstrated that an 8-week rehabilitation program focusing on strengthening and neuromuscular control of hip and core muscles led to positive patient outcomes, improved hip and core strength, and reduced knee abduction motion influenced by PFPS. During 2019 to 2021, Crossley, KM et al^[7]^ established a scientific framework for PFPS clinical practice through the 4th International Patellofemoral Pain Consensus Statement, covering terminology, definitions, clinical examination, natural history, patellofemoral osteoarthritis, and patient-reported outcomes. From 2020 to 2023, Smith, BE et al^[3]^ conducted a systematic review and meta-analysis on the incidence and prevalence of patellofemoral pain, revealing high rates – with an annual prevalence of 22.7% in the general population and 28.9% in adolescents. Given the poor long-term prognosis and significant disability associated with PFPS, the authors emphasized that it should be an urgent research priority. In the same period, Collins, NJ et al^[34]^ published a consensus statement on exercise therapy and physical interventions (orthoses, taping, and manual therapy) for patellofemoral pain. The expert panel recommended combined hip and knee-focused exercise therapy, multimodal interventions, and foot orthoses to improve pain and function. The use of electrophysical modalities alone was not advised, while uncertainty remains regarding patellar taping/bracing, acupuncture/dry needling, soft tissue manual therapy, blood flow restriction training, and gait retraining. This statement has helped standardize clinical practice and encouraged further research in PFPS. From 2022 to 2025, the clinical guidelines for PFPS by Willy, RW et al^[22]^ synthesized current evidence to support clinical management. These guidelines and consensus statements have been widely read and cited in the field. They summarize the state of PFPS research, guide clinical researchers, and reflect the growing emphasis on evidence-based practice, ethics, and patient safety.

### 4.3. Research frontiers in PFPS

In bibliometric analysis, keyword clustering helps reveal research hotspots and thematic distributions, constructing the knowledge structure of a field. As shown in Figure 9A, current research hotspots include knee, kinematics, pain, reliability, hip, biomechanics, osteoarthritis, strength, females, and rehabilitation. Figure 9B displays 4 major clusters in PFPS research. Among these, the green cluster focuses on the pathogenesis, diagnosis, and surgical treatment of PFPS, including keywords such as replacement, total knee arthroplasty, reconstruction, follow-up, mri, and outcomes. The blue cluster emphasizes conservative treatment and functional rehabilitation, including muscle strength, physiotherapy, exercise, quality, and disability. The pink cluster represents biomechanical aspects of PFPS, containing kinematics, mechanics, motion, hip strength, running, and walking. Future research should integrate biomechanics, neuroscience, and rehabilitation medicine to establish a holistic anatomy-function-rehabilitation framework, which appears to be an emerging research priority. In addition, other closely connected keywords form smaller clusters, such as the yellow cluster, which concentrates on neurophysiology in PFPS, including electromyography, activation, and performance.

Keyword bursts reflect frontier topics and research frontiers. In our analysis, currently emerging burst keywords originate from 3 distinct domains. The first category relates to interventions and treatments, including therapy, program, and single-leg squat, highlighting specific therapeutic methods and program design. The second category involves biomechanics and movement patterns, including walking, movement system, knee kinematics, trunk, and patterns, aiming to explain underlying mechanisms through motor control and joint kinematics. The third category focuses on research methodology, particularly randomized controlled trials, underscoring the importance of evidence-based medical research. Future efforts should strengthen the evidence from randomized controlled trials while continuing to develop systematic reviews.

### 4.4. The pathogenesis, diagnosis, and surgical treatment of PFPS

The pathogenesis of PFPS currently has no definitive conclusion. Clinicians believe that PFPS is caused by abnormal patellar tracking motion, which leads to excessive compressive stress on the patellar surface. Factors that may cause abnormal patellar tracking motion include quadriceps weakness, quadriceps muscle imbalance, excessive tightness of knee soft tissues, increased quadriceps angle (Q angle), hip weakness, and altered foot kinematics.^[1,35]^ Q angle measurement is obtained by measuring the angle between the line connecting the anterior superior iliac spine to the center of the patella and the line connecting the tibial tubercle to the center of the patella.^[36–38]^ In addition, some studies suggest that its pathogenesis includes internal factors, such as skeletal alignment, soft tissue imbalances, and biomechanical influences, as well as external factors such as environment and equipment.^[7–9]^

A key point in diagnosing PFPS is that the patient’s pain worsens after running, kneeling, squatting, or prolonged sitting with the knee bent,^[7,22,36,39–41]^ and sometimes a small amount of effusion may occur, although this is not common.^[7,36]^ Physical examination tests that can be performed to diagnose PFPS include Q angle measurements, J sign, patellar glide, patellar tilt test, patellar grind/Clarke test, Ely test, and double-/single-leg squats. Research has proven that pain during double-leg squats is the most sensitive physical examination finding for PFPS and should always be used as a functional diagnostic test during evaluation.^[7,22,40,42]^ In addition, musculoskeletal ultrasound can assist in diagnosing PFPS. PFPS may present with intra-articular effusion, quadriceps tendon thickness ≥0.54 cm, patellar tendon thickness ≥0.35 cm, asymmetry in gluteus medius thickness during contraction, as well as smaller vastus medialis muscle volume, insertion level, and fiber angle. However, imaging is typically not used for initial diagnosis of PFPS unless there is a history of trauma or dislocation.^[36,43]^ Diagnosticians should also differentiate from other diseases such as patellofemoral osteoarthritis, prepatellar bursitis, quadriceps tendinopathy, Osgood Schlatter disease, and referred pain from the ipsilateral hip to avoid misdiagnosis.^[22]^

Surgery is generally considered a last resort treatment, with many experts recommending trying at least 24 months of conservative treatment first. If surgery is needed, it may include removing damaged articular cartilage of the patella and performing a lateral retinacular release so as to allow optimized tracking of the patella in the trochlear groove.^[44]^ However, recent studies report that surgical treatment for PFPS provides no additional benefit compared with conservative treatment.^[45]^

### 4.5. Conservative treatment and rehabilitation of PFPS

Conservative treatment is the preferred approach for managing PFPS, including exercise therapy, patellar taping, foot orthoses, manual therapy, electrotherapy, biofeedback, and pharmacological interventions.^[46,47]^ The expert panel recommends the use of exercise therapy, combined interventions, and foot orthoses to improve pain or function in individuals with patellofemoral pain.^[34]^

According to the 2016 consensus statement from the International Patellofemoral Pain Research Association, exercise therapy is the “preferred treatment” for patients with PFPS, with high-quality evidence supporting its effectiveness in improving pain and function in the short, medium, and long term. Exercise therapy is the only intervention to receive such a strong recommendation.^[7]^ Exercise therapy includes open kinetic chain exercises and closed kinetic chain exercises. Open kinetic chain exercises include straightening the leg and raising it up, quadriceps strengthening, knee extensions, and hip abduction. Closed kinetic chain exercises include sitting against the wall, double-leg and single-leg squats, lateral step-downs, and leg presses. If patients demonstrate deficits or imbalances in these areas, strengthening exercises for the core and ankle musculature should also be included.^[48,49]^

Among the top 10 most published authors, Rathleff, MS, Crossley, KM, and Vicenzino, B, among others,^[50]^ collaborated on a randomized clinical trial that directly compared the efficacy of foot orthoses and hip exercises for treating PFPS. Participants receiving orthoses were also required to perform a daily home-based foot and ankle exercise program twice a day for 6 weeks.^[51]^ The program included stretching of the triceps surae/Achilles tendon complex for 30 seconds per repetition, repeated 3 times, as well as foot anti-pronation exercises. Participants in the progressive hip resistance exercise group performed hip-focused exercises 3 times per week for 4 weeks, targeting the hip abductor, extensor, and external rotator muscle groups bilaterally. Resistance was provided using elastic bands, with 10 repetitions per exercise and 90-second intervals between sets. The Global Rate of Change Scale was used as the primary outcome measure, while the Numeric Pain Rating Scale, the Knee Injury and Osteoarthritis Outcome Score, and midfoot mobility, among others, served as secondary measures. The trial demonstrated that hip exercises were more effective than foot orthoses, and that midfoot mobility was more closely associated with the foot orthoses intervention compared with hip exercises.

In the author collaboration network, Crossley, KM, Vicenzino, B, and Collins, NJ, among others from Australia,^[52]^ conducted a randomized controlled trial in which adolescents aged 12 to 18 with PFPS received either foot orthoses or flat shoe inserts over a 3-month period. Knee-related pain, symptoms, function, and quality of life were assessed at 6 weeks and 3 months. The results indicated that soft foot orthoses combined with an exercise program significantly reduced PFPS symptoms compared with flat inserts and exercise alone. Crossley, KM and Collins, NJ, among others,^[53]^ compared the efficacy of foot orthoses and flat shoe inserts, evaluating pain levels using visual analogue scale and Global Rate of Change Scale at baseline and after 6 weeks of treatment. They found that both groups showed significant improvements in pain, but the foot orthosis group experienced greater pain reduction, possibly due to the arch contour of the orthoses reducing peak plantar pressure.^[54]^ These studies suggest that foot orthoses are one of the effective therapies for alleviating symptoms and pain in PFPS and that combining foot orthoses with exercise therapy may yield better outcomes.

### 4.6. Overall view of the motion chain

Biomechanics is also an important research focus in studies on PFPS. As a common sports-related injury,^[4,5]^ PFPS has naturally attracted attention in the fields of sports science and biomechanics. Abnormal biomechanics is one of the risk factors that cause runners to develop PFPS.^[8–10]^

We have observed that biomechanical research on PFPS has gradually expanded from initially focusing on the bone and muscle structures around the knee to considering the entire lower limb movement chain as a whole. This shift can also be seen in the phase-specific hotspots shown by the timeline viewer of CiteSpace software. Starting from 2005, research mainly focused on the knee area, with hot keywords such as patella joint, knee joint, and anterior cruciate ligament. After 2010, keywords such as lower extremity, lower extremity kinematic, gait, and hip strength gradually emerged. Researchers have gradually discovered a close relationship between abnormal hip function and patellofemoral pain syndrome. The patellofemoral joint is very sensitive to this specific hip condition, meaning that in some patients, the root cause of PFPS may not be the patellofemoral joint itself.^[55]^ However, there are few articles in this area, and more evidence is needed to support this view in the future. Other studies show that in most functional activities, there is no difference in gluteus medius and gluteus maximus activation levels between the PFPS group and the healthy group. Regarding kinematic changes, moderate evidence indicates that during running and single-leg squat activities, the PFPS group shows increased peak hip adduction. Most importantly, strong evidence shows that compared with the control group, PFPS patients have weaker hip strength, smaller hip external rotation and abduction strength, and less use of hip external rotator and abductor muscles.^[56]^ In addition, the calculated signal variation coefficient for hip extension in PFPS is significantly higher, indicating greater signal noise and thus poorer neuromuscular control of the hip extensor muscles.^[57]^ Rehabilitation training can improve this problem. Research by Earl, JE et al^[33]^ shows that an 8-week rehabilitation program focusing on strengthening and improving neuromuscular control of the hip and core muscles can lead to positive patient outcomes, increase hip and core muscle strength, and reduce knee abduction movement caused by PFPS progression. Furthermore, Bartsch, A et al^[58]^ studied the biomechanical and biological factors of sexual dimorphism in PFPS. They found that on average, women have a smaller patella, higher patellofemoral cartilage stress, and less favorable trochlear shape, ligament and muscle composition, and neuromuscular control patterns for preventing PFPS. In comparison, men show stronger hip external rotation ability on average, which can help prevent PFPS. Especially in kinetic and kinematic analysis, the risk factor characteristics in men are clearly different from those in women. Sex hormones may also play a role in PFPS risk: estrogen may cause ligament looseness, increase midfoot load, and affect lower limb neuromuscular control, while testosterone has a positive effect on muscle mass and strength.

### 4.7. Limitations

Our search was limited to the WoSCC. Although it is considered one of the most authoritative databases, it does not include all relevant articles from other academic databases such as PubMed and Scopus. Therefore, our results may not fully represent the entire field of PFPS research. In addition, we only included English-language articles and review articles, which may have led to the omission of important studies published in other languages. In this study, we analyzed all keywords, including author keywords and additional keywords added by database editors based on the article topics. As author keywords are always chosen by the authors themselves, they may not always accurately represent the research focus of the paper and could sometimes be misleading.

## 5. Conclusion

In conclusion, we conducted a bibliometric analysis to analyze the research status and hotspots in the field of PFPS over the past 2 decades. The United States contributed the highest number of publications (333 articles). Both the annual publication count and average citation rate showed an overall upward trend; most publications were published in 2022, and the highest average number of citations was in 2019. The most cited article was “Patellofemoral pain” by Willy, RW, with a total of 182 citations. The *International Journal of Sports Physical Therapy* published the most articles in this field, while the University System of Ohio and Rathleff, MS were the most productive institution and author, respectively. Collaboration network analysis revealed close cooperation among scholars from leading countries and institutions.

Current research hotspots focus on the knee, kinematics, anterior knee pain, hip, biomechanics, reliability, pain, osteoarthritis, strength, and females. Keyword burst suggests that future developments may center on knee kinematics and patellar dislocation. The field of PFPS is expected to continue evolving, with interdisciplinary and innovative approaches further promoting our understanding and management of the condition.

## Author contributions

**Conceptualization:** Siliang Zeng.

**Data curation:** Wen Zheng, Siliang Zeng.

**Formal analysis:** Wen Zheng, Siliang Zeng.

**Investigation:** Wen Zheng.

**Methodology:** Wen Zheng, Siliang Zeng.

**Software:** Wen Zheng.

**Project administration:** Siliang Zeng.

**Resources:** Siliang Zeng.

**Supervision:** Siliang Zeng.

**Validation:** Siliang Zeng.
